# Attitudes toward and regional utilization of age-specific vaccinations from the age of 60 in Saxony-Anhalt

**DOI:** 10.1007/s43999-025-00079-9

**Published:** 2025-12-03

**Authors:** S. Nestler, S. Walter, I. Hrudey, C. Hasenpusch, J. Holstiege, J. Bätzing, S. March, E. Swart, C. Stallmann

**Affiliations:** 1 Institute of Social Medicine and Health Systems Research Faculty of Medicine, Otto-von-Guericke University Magdeburg, Magdeburg, Germany; 2 Department of Social Work, Health and Media, Magdeburg-Stendal University of Applied Sciences, Magdeburg, Germany; 3 Central Research Institute of Ambulatory Health Care in Germany (Zi), Berlin, Germany

**Keywords:** Saxony-Anhalt, Elderly vaccination, Prevention, Influenza, Pneumococcus, Herpes zoster

## Abstract

**Background:**

Saxony-Anhalt (ST) has the oldest population in Germany, with partly above average and increasing prevalences of chronic diseases and multimorbidity. Vaccinations are crucial to prevent infections and severe disease outcomes in older adults, especially in rural areas. However, national immunisation recommendations for individuals aged 60 and above are only partially followed. This research aims to examine (1) actual vaccination utilization and (2) the determinants, reasons, and barriers to vaccination uptake in ST’s elderly population (≥ 60 years).

**Methods:**

(1) Vaccination utilization data from 760,868 individuals (aged ≥ 60 years ) for influenza, pneumococcus, and herpes zoster from 2011 to 2020 were analyzed using outpatient care data from the Central Research Institute for Outpatient Health Care in Germany (Zi). (2) Additionally, subjective vaccination behaviors, attitudes, and barriers were surveyed from 864 participants in two urban and two rural municipalities as part of the “Prevention in Old Age in Saxony-Anhalt” (PrimA LSA) project.

**Results:**

The secondary data set included 760,868 individuals for influenza, pneumococcal, and herpes zoster vaccination. Immunisation rates for influenza and pneumococcus in ST were higher than the national average, but rates for herpes zoster were lower. A survey of 864 participants over 60 revealed that the subjective vaccination rate was overestimated. Key barriers included lack of information and limited access to vaccination services. The study also reaffirmed the central role of general practitioners in healthcare delivery.

**Conclusion:**

Despite positive attitudes towards vaccination, targeted educational initiatives are necessary to increase vaccination rates. General practitioners need additional support, and improving health literacy in the elderly is crucial to enhance vaccine acceptance and disease prevention.

## Introduction

Germany’s population is undergoing a process of ageing, as indicated by the increasing average age across the country. The federal state Saxony-Anhalt (ST), with an average age of 48.1 years, has the oldest population in Germany [1]. In 2021, the proportion of over 65-year-olds reached a new high of 27.6% [2]. The demographic change is accompanied by an increasing prevalence of chronic diseases and multimorbidity, a trend that will lead to further long-term challenges – especially in the medical care [1–5].

Vaccinations are essential for preventing infectious diseases that increase in incidence with age or cause severe outcomes in older adults [6–8]. Herpes zoster incidence rises substantially after age 60 due to immune senescence [9], while influenza and pneumococcal infections account for 70–85% of seasonal flu-related deaths in individuals aged ≥ 65 years [10, 11]. Effective vaccination coverage is therefore critical to reduce morbidity and mortality in this population [6].

The implementation of preventive health services, such as vaccinations, plays a pivotal role in meeting these needs [12]. According to the German Standing Committee on Vaccination (STIKO), individuals aged 60 and above are advised to receive influenza vaccinations on an annual basis, herpes zoster vaccinations (which may be administered in two combined doses) and a single pneumococcal vaccination (to commence at the age of 60) illustrated in Table 1 [13]. Individuals up to 60 years are recommended vaccinations only by specific medical indications (e.g. chronic morbidity).

**Table 1:** STIKO vaccination recommendations for individuals aged 60 and older [14]

Vaccine	Year of Introduction	STIKO-Recommendation since
Influenza	1960s (GDR), 1982 for the chronically ill, all persons ≥ 60 years and medical personnel (FRG) 2002 for persons >60 years	From 1982 for the chronically ill, all persons ≥ 60 years of age and medical staff Standard vaccination for all persons ≥ 60 years; indication vaccination for chronically ill persons and medical personnel From 2010 extension of the indication to pregnant women
Pneumococci	2001 with polysaccharide for persons >60 2006 for all children 2023 with 20-valent conjugate vaccine for persons >60	From 2001 single standard vaccination with a polysaccharide vaccine for persons aged ≥ 60 years From 2006 all children up to 24 months 2016 update Indication vaccination of risk groups (sequential vaccination) and senior citizens 2023 Update Single standard vaccination ≥ 60 years with 20-valent conjugate vaccine (PCV20)
Herpes Zoster	2018	Since 2018 two-dose regimen for full immunity ≥ 60 years

Note: Prior Until 1990, Germany was devided into GDR (German Democartic Republic) and the FRG (Federal Republic of Germany), each with its own vaccination policies. Following, STIKO recommendations became valid nationwide

ST achieved a vaccination rate of 67.5% in the 2020/21 influenza vaccination season [15], significantly exceeding the national average of 47.3% and surpassing rates in other federal states. However, the WHO’s target of 75% vaccination coverage was still not met [16]. This relatively high uptake in ST makes it a valuable case for further investigation. Recent research from the federal state of Thuringia demonstrated that repeated influenza vaccination among adults aged 60 and above is influenced by a variety of factors, including age, institutional care, underlying health conditions, and participation in disease management programs [8]. These findings underscore the need for regionally specific analyses combining claims and survey data to comprehensively understand vaccination behavior in older adults. While routine data provide insights into structural and medical factors (e.g., healthcare infrastructure, physician density), survey data capture individual-level determinants such as attitudes, perceived barriers, and personal motivations. The example of high uptake rates in ST provides an opportunity to leverage this combined approach, examining both objective vaccination patterns and subjective perspectives across different municipalities and age groups aged 60 and over. The results allow for the derivation of targeted implementation strategies to enhance uptake of age-specific vaccinations.

This study addressed three research questions: (1) How does self-reported vaccination uptake compare to actual vaccination rates among individuals aged ≥ 60 years in Saxony-Anhalt? (2) What regional variations exist in vaccination uptake? (3) What barriers and facilitators influence vaccination behavior?

## Methodology

The data collected via a multimethod approach conducted by the project ‘Prevention in Old Age in Saxony Anhalt’ (PrimA LSA) in the target group of citizens aged over 55 years in ST, consistent with STIKO vaccination recommendations for this high-risk age group.

### Resident survey (PrimA LSA)

The cross-sectional survey was conducted between 21 April and 8 June 2021 as part of the ‘Prevention in Old Age in Saxony-Anhalt’ (PrimA LSA) project, funded by the European Regional Development Fund (ERDF) and ST (IDs: ZS/2019/07/99610, ZS/2020/06/145442). Four municipalities were selected to capture ST’s demographic and socioeconomic heterogeneity [17]: Halle (Saale) and Magdeburg as urban centers, and Wanzleben-Börde and Sangerhausen representing rural regions. Due to the lack of comprehensive population registries in the Börde and Mansfeld-Südharz districts, the cities of Wanzleben-Börde and Sangerhausen were chosen as proxies, closely matching their respective counties’ profiles [17].

From a gross sample of 3,665 invited residents aged ≥ 60 years (including private households and long-term care facility residents), 954 completed the written postal survey (26% response rate). The detailed procedure is outlined in the study protocol [17]. Although the original dataset included individuals aged 55–59 years, this analysis focuses exclusively on those aged ≥ 60 years, aligning with STIKO recommendations.

### Claims data (Zi)

Anonymized aggregated claims data on outpatient medical services were obtained from the Central Research Institute of Ambulatory Health Care in Germany (Zentralinstitut für die kassenärztliche Versorgung, Zi) for the period 2011–2020. These data originate from routine billing coded according to the German Uniform Evaluation Standard (Einheitlicher Bewertungsmaßstab, EBM), submitted by physicians to Regional Associations of SHI Physicians (§ 295 SGB V) and subsequently aggregated by the Zi The data were subsequently transferred to the Zi, where they were aggregated. The data represent a complete census of all SHI-insured individuals aged ≥ 60 years in ST with at least one outpatient physician contact, covering the vast majority of the older population. Since the Zi compiles national data from all 17 regional associations of SHI physicians in Germany, ST utilization rates could be compared against the German national mean.

### Variables

#### Resident survex variables

The survey assessed self-reported vaccination status and factors influencing vaccination attitudes. Barriers included subjective attitudes toward vaccination [18], fears of side effects, and access obstacles [19]. Facilitators encompassed physician recommendations [19] and active vaccination reminders from healthcare providers [20].

#### Claims data variables

Vaccination uptake was assessed for STIKO-recommended vaccines: influenza, pneumococcal disease, and herpes zoster. Influenza vaccination was measured per season (Q3 of one year through Q1 of the following year) to reflect its seasonal nature, while pneumococcal and herpes zoster vaccinations were calculated annually. EBM codes used are detailed in Table 2. The analysis was stratified by age groups (5-year intervals), sex, region (district and urban district levels), and where data permitted health status (presence of chronic conditions with vaccination indications, identified via ICD-10 codes) using established risk stratification algorithms [21–23].

**Table 2:** EBM codes (GOPs) for vaccinations analysed in individuals aged 60 and older

Vaccine	EBM code	Explanation
Influenza	89,111 89,112	Annual vaccination from age 60 Annual vaccination for high-risk groups
Pneumococci	89,119 89119R, 89,120	Standard vaccination from age 60; Vaccination for indication-based cases
Herpes Zoster	89,128 A/B 89,129 A/B	Basic immunisation from age 60 for high-risk groups

### Statistical analysis

For the resident survey, descriptive statistics and normality tests were performed using IBM SPSS Statistics 26 (Armonk, NY, USA). Kolmogorov-Smirnov tests assessed variable normality. Mann-Whitney U tests were employed for ordinal or non-normally distributed interval-scaled variables, and Kruskal-Wallis tests for nonparametric comparisons across more than two groups.

For claims data, given the complete census of SHI-insured individuals, vaccination uptake was analyzed descriptively on a cross-sectional basis for each calendar year (2011–2020), without longitudinal follow-up. ST vaccination rates were compared against the German national mean using absolute differences. Significance tests were not performed, as census-level sample sizes render p-values uninformative for clinical interpretation.

## Results

### Actual vaccination uptake (Zi data incl. regional comparison)

**Fig. 1:**
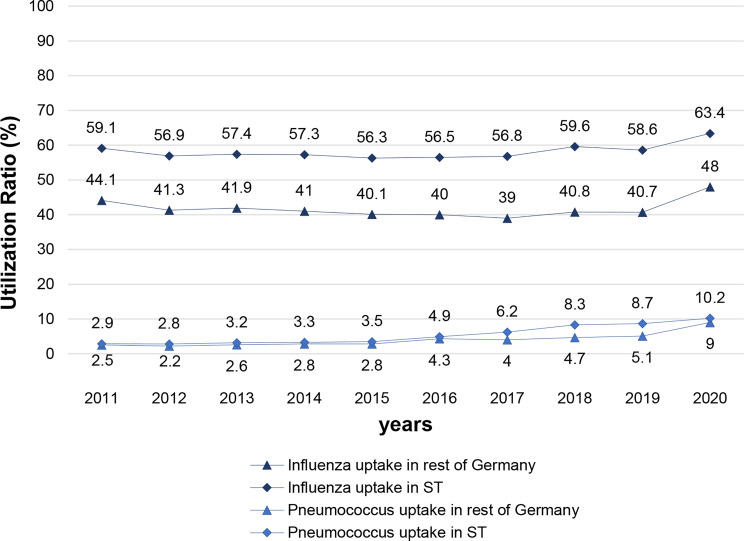
Actual uptake of the influenza and pneumococcal vaccination of over-60s in the rest of Germany and in Saxony-Anhalt (ST) as a whole from 2011 to 2020 based on the Zi data in per cent (%), Note. Sample data available in Appendix A

The characteristics of the sample due to age, sex and region is shown in Appendix A. Figure 1 illustrates the 10-year trend in vaccination rates in Saxony-Anhalt, from 2011 to 2020. Overall, the influenza vaccination was taken up by more than half of the over-60s in Saxony-Anhalt. In 2020, in particular, the vaccination rate (63.4%) rose by approximately 5% compared to the previous year (58.6%). In all years, the vaccination rate of Saxony-Anhalt (63.4%) was consistently higher than in the rest of Germany, reaching a 15.4%-points higher vaccination rate in 2020 compared to the national average of 48.0%.

The uptake of the pneumococcal vaccinations was significantly lower than that observed for the influenza vaccination. The uptake was below 10 per cent in 2019. This lower uptake is consistent with the fact that pneumococcal vaccination is typically recommended as a one-time immunization, as detailed in Table 1. Nethertheless the pneumococcal vaccination rate was consistently higher than that observed for the rest of Germany. Notably, between 2017 and 2019, vaccination rates in Saxony-Anhalt consistently exceeded those in the rest of Germany, with differences ranging from approximately 2.2% to 3.6%. These years marked the period with the most pronounced disparities in regard to the pneumococcal vaccination rate compared to the national average.

The vaccination against herpes zoster has the lowest uptake of all recommended vaccinations for the over-60 age group, with a vaccination rate of less than 5%. Specifically, uptake in the rest of Germany increased from 0.9% in 2019 to 2.7% in 2020, while in Saxony-Anhalt it rose from 0.8% to 4.2% during the same period. This represents a five-fold increase in uptake compared to the previous year. Specifically, uptake in Saxony-Anhalt increased from 0.8% in 2019 to 4.2% in 2020, while in the rest of Germany it rose from 0.9% to 2.7% during the same period. This represents a five-fold increase in uptake compared to the previous year. It is important to note that an adjuvanted herpes zoster subunit vaccine (Shingrix) recommended by the STIKO has only been available in Germany since March 2018, as shown in Table 1. Consequently, data are only available from 2019 [24]. To enhance clarity and streamline presentation, these results are not included in Fig. 1.

The uptake rates for the three recommended vaccinations (Influenza, Pneumococcal, and Herpes Zoster) indicate that the Influenza vaccination was the most commonly administered vaccine in both Saxony-Anhalt and the rest of Germany. In regard to gender differences in 2020 for the Influenza vaccination, the uptake in Saxony-Anhalt was 61.8% for men and 63.6% for women (overall: 62.7%), while in the rest of Germany, men and women had nearly identical rates of 47.6% (overall: 47.6%). The Pneumococcal vaccination followed a similar pattern. In Saxony-Anhalt, the uptake was 10.0% for men and 10.4% for women (overall: 10.2%), while in the rest of Germany, the rates were identical for men and women at 8.9% (overall: 8.9%). For the Herpes Zoster vaccination, the uptake was slightly higher among women than men in both Saxony-Anhalt and the rest of Germany. In Saxony-Anhalt, the rates were 3.6% for men and 3.9% for women (overall: 3.75%), whereas in the rest of Germany, they were 2.4% for men and 2.8% for women (overall: 2.6%). In summary, gender-specific differences in vaccination uptake were small but consistent, with women exhibiting slightly higher rates than men across all three recommended vaccines.

### Regional uptake

**Fig. 2:**
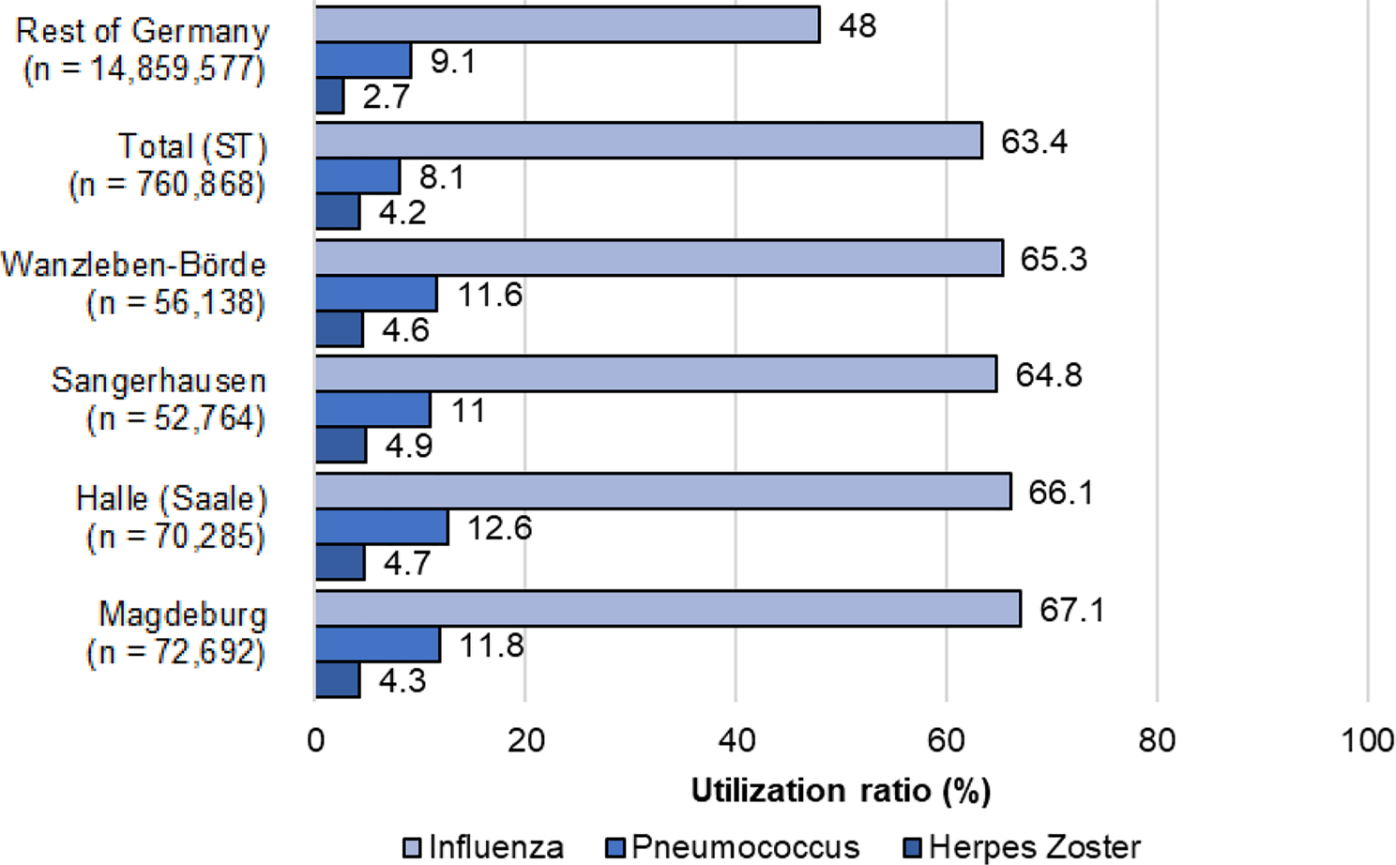
Actual uptake of influenza, pneumococcal, and herpes zoster vaccination among those over 60 years of age in regional and nationwide comparison in 2020 based on Zi data, percent (%), *N* = 15,620,445

At the regional level of ST, a higher uptake rate can be observed in comparison to the average across the federal state. In the urban municipalities of Halle and Magdeburg, there is an increased coverage of influenza and pneumococcal vaccination in comparison to the more rural municipalities. In contrast, the use of herpes zoster vaccination is again higher in the rural municipalities. Only data from 2020 were used for this comparison, as vaccination uptake rates have shown only slight increases in recent years. An overview is shown in Fig. 2.

### Subjective vaccination utilisation of the resident survey

**Fig. 3:**
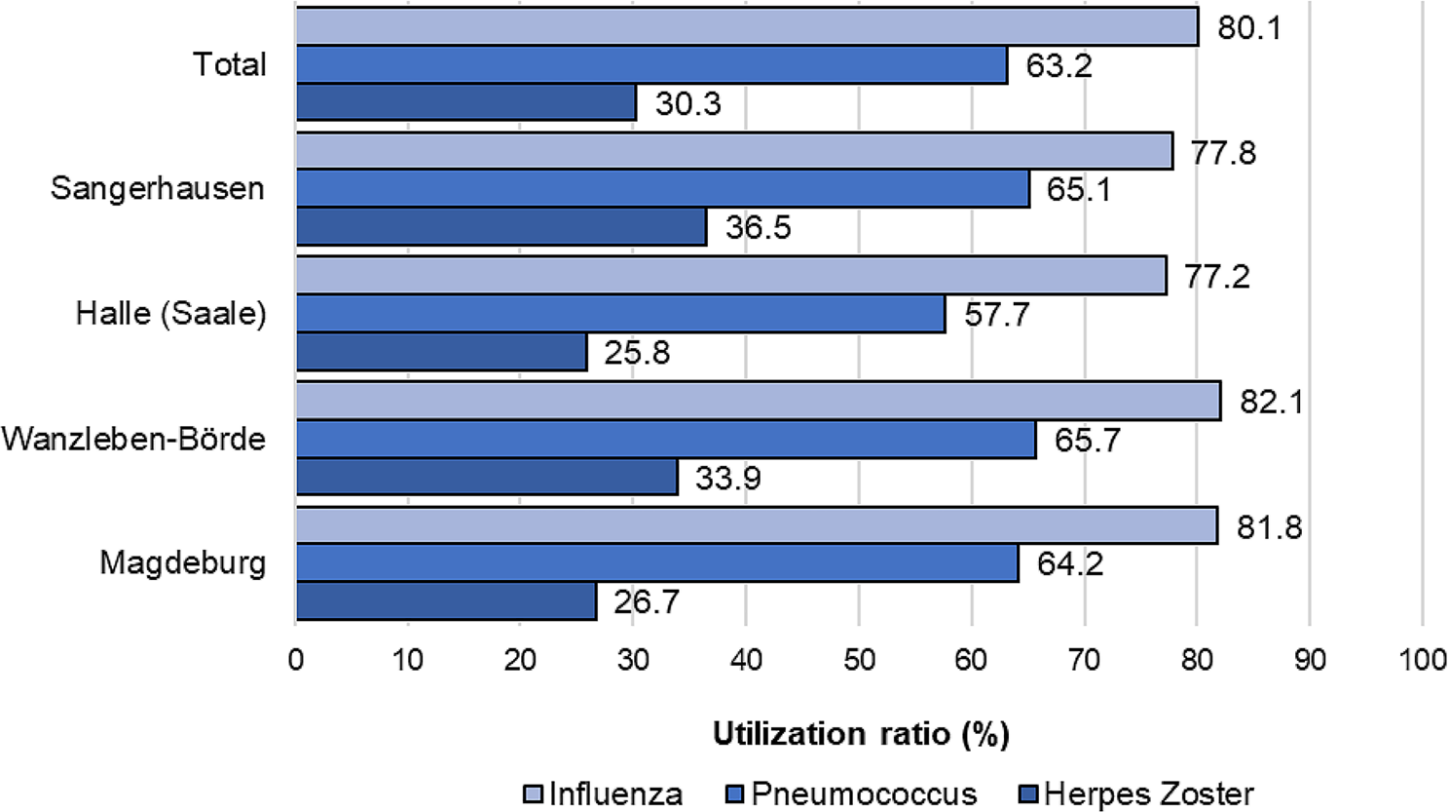
Subjective, ever-use of influenza, pneumococcal and herpes zoster vaccination of the over-60s in total ST and the four municipalities of the residents’ survey in percent (%), *N* = 864

The characteristics of the sample is shown in Appendix B. The majority of the over-60s surveyed (87.3%) stated that they had already received influenza vaccinations. Of these, 74.5% said they had even received influenza vaccination annually. Two-thirds said they had already received the recommended pneumococcal vaccination and slightly less than one-third (30.1%) the herpes zoster vaccination (Fig. 3). Detailed regional vaccination rates by district and year are provided in Appendix C.

A regional comparison also shows that the herpes zoster vaccination was taken up significantly less frequently in the Region of Halle (Saale) (5.9%) and Sangerhausen (7.5%). With regard to the uptake of influenza and pneumococcal vaccination, the subjective uptake rate in northern Magdeburg was in some cases more than 10% points higher than in the other three municipalities. No statistically significant differences in vaccination uptake across the four study regions.

#### Influence of vaccination attitude and fear of side effects on subjective vaccination uptake

#### Attitude towards vaccination in general

The majority (84.7%) of respondents expressed a very positive attitude towards vaccination and reported that they usually received the recommended vaccinations. A further 12.6% of respondents did not take advantage of individual vaccinations, but did not reject them. Only 2.7% of respondents had a negative attitude towards some vaccinations, while 0.1% had a negative attitude in general. A comparison of the groups according to their general attitudes towards vaccination revealed significant differences in the uptake of the three vaccinations considered here (*p* < 0.001).

#### Attitude towards side effects

The majority of respondents (56.7%) stated that they were not afraid of possible side effects from a vaccination in general. A further 30.1% indicated that they were only slightly afraid. A divided opinion was held by 10.1% of respondents, with 1.9% stating that they were somewhat afraid and 0.5% indicating that they were very afraid. Female respondents were significantly more likely to report fear of side effects (*p* < 0.001). This suggests that a favourable vaccination attitude also contributes to a higher vaccination uptake. Similar results were obtained when considering the influence of fear of side effects on the uptake of the recommended vaccinations. Influenza and pneumococcal vaccination were taken up significantly more often by people with no or low anxiety (p_Influenza_ < 0.001; p_Pneumococci_ = 0.031). Only the uptake of the herpes zoster vaccination was not significantly affected by the sense of fear.

#### Reasons for and barriers to subjective vaccination uptake

**Fig. 4:**
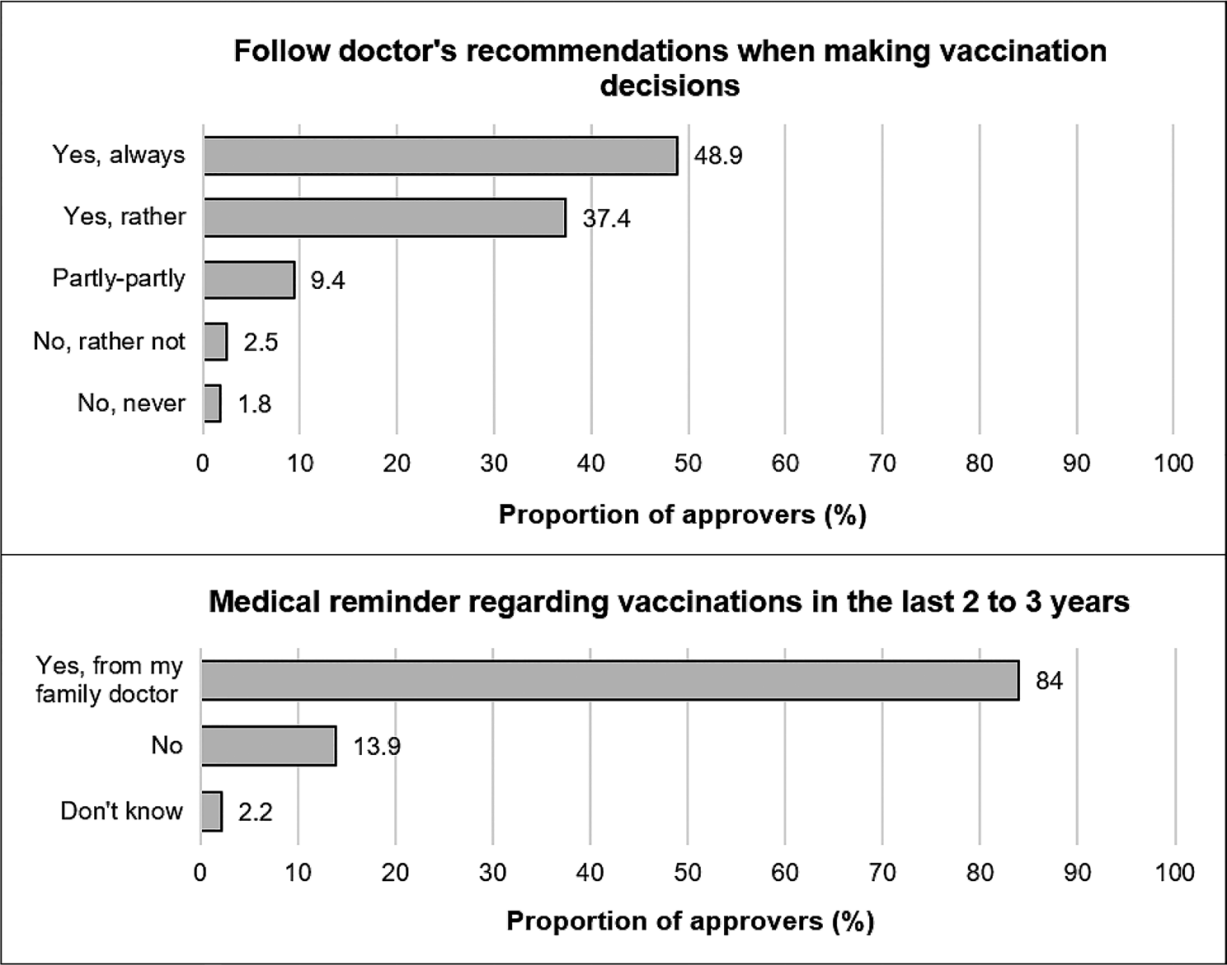
Consideration of medical recommendations and reminders for vaccination uptake in 2021, *N* = 864

The majority of respondents aged 60 and above who were surveyed indicated that they either always (48.9%) or typically (37.4%) adhere to medical recommendations when making decisions about vaccinations. Similarly, the majority of respondents in this age group reported that they either always (48.9%) or frequently (37.4%) follow their doctor’s recommendations regarding vaccinations. Furthermore, 84% of respondents indicated that their general practitioner (GP) had provided them with a reminder of recommended vaccinations within the past two to three years. Only 13.9% of respondents reported that they had not received such a reminder (Fig. 4).

**Fig. 5:**
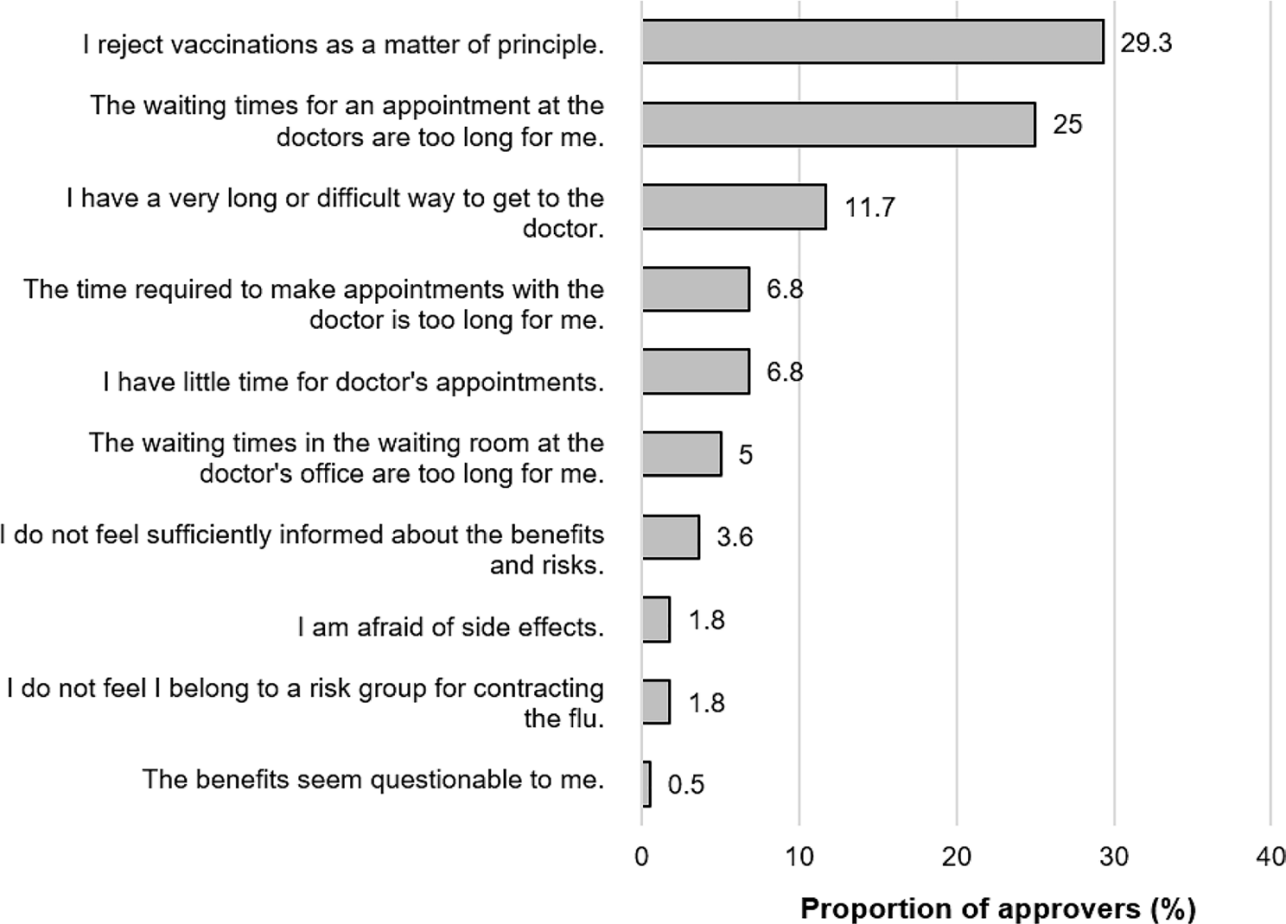
Self-reported Barriers to the uptake of influenza vaccination among over-60s Participants in the residents’ survey in percent (%), 205 multiple responses from 169 cases

Self-reported influenza vaccination uptake was significantly higher among respondents who followed physician recommendations (mean rank = 463.2) compared to those who did not (mean rank = 311.0, *p* < 0.001). Physician reminders in the last 2–3 years also showed a significant association with vaccination uptake (χ² = 54.1, df = 2, *p* < 0.001), with higher uptake among those receiving reminders (mean rank = 395.4) versus those without (mean rank = 495.5). To identify inhibiting factors for uptake, Fig. 5 illustrates potential barriers to uptake using the example of influenza vaccination. Sex-stratified analyses revealed no significant differences.

Among unvaccinated respondents (*N* = 222), barriers to influenza vaccination were assessed (Figure X). The most frequently cited barriers were principled rejection of vaccination and appointment-related waiting times. Access and time constraints, including travel difficulties and scheduling challenges, were reported by smaller proportions of respondents. Information-related concerns and fear of adverse effects were relatively uncommon. Only minimal proportions questioned vaccine benefits or perceived themselves as outside risk groups. Sex-stratified analyses revealed no significant differences in reported vaccination barriers between men and women.

## Discussion

The Zi data, which covers all statutory insured insures in ST and about 95% of the total population, indicates that over half of the over-60s in ST were vaccinated against influenza. In contrast, uptake of the pneumococcal and herpes zoster vaccinations was significantly lower. The influenza vaccination uptake among individuals aged 60 years and above in Germany during the 2020/21 season was approximately 47.3% [25]. The uptake of vaccinations against herpes zoster (nationwide 3.3% − 5.0% in the first quarter of 2019–2021) and pneumococcal (nationwide 22.5% in the first quarter of 2021) was considerably lower in this target group [25].

Moreover, the data revealed that ST exhibited the highest vaccination rates (in relation to influenza, pneumococci and herpes zoster) in a nationwide comparison. This can be attributed to the considerably higher willingness to vaccinate in the eastern compared to the western federal states reflection vaccination experiences in former GDR [26]. Statistical analyses revealed no significant differences in vaccination uptake between the four study municipalities. Furthermore, the study revealed that there were minimal differences between urban and rural municipalities in terms of vaccination uptake when compared on a regional level. This may be attributed to the socialisation experienced in the German Democratic Republic (GDR), which included compulsory examinations and school and company vaccinations [27]. However, the relative homogeneity within Saxony-Anhalt may also reflect regional healthcare infrastructure factors. Eastern German regions, particularly Saxony-Anhalt, face substantial aging of the primary care workforce with insufficient young physician recruitment, affecting nearly all districts [28], compounded by outmigration to western states [29] and rural care disparities [30].

The observed rise in vaccination rates in 2020 may be attributable to heightened awareness during the COVID-19 pandemic. In Germany, influenza vaccination coverage among adults aged ≥ 60 years increased significantly from 38.8% (2019/2020) to 47.3% (2020/2021) [31]. Saxony-Anhalt demonstrated the highest influenza vaccination coverage nationwide (61.3% in 2021/2022) [31]. Sustaining this increased vaccination awareness requires continued targeted interventions, particularly in rural areas.

In contrast to the actual vaccination uptake, the subjective rate was clearly overestimated for all vaccinations. This can be attributed to the fact that the sample was extremely fit and healthy. In the case of the residents’ survey, there is a bias due to the predominant participation of more health-conscious people who are interested in the topic anyway [32, 33]. It can also be assumed that more socially desirable answers were given that do not correspond to actual vaccination behaviour. Furthermore, the use of pneumococcal and herpes zoster vaccinations was not surveyed for the specific year or a potential booster. Consequently, in addition to the interesting comparison between self-perception and perception by others, it should be noted that the results of the resident survey are not representative.

Nevertheless, the results of the resident survey provided far-reaching information about the opportunities and barriers to the uptake of the vaccinations under investigation. The results of the resident survey demonstrated a high level of willingness to be vaccinated among those surveyed who were over the age of 60. A positive attitude towards vaccinations is a significant predictor of their uptake [34, 35]. Further studies have demonstrated that the proportion of individuals with positive attitudes towards vaccination has increased in all education status groups by 2018 [36].

A study involving 103,163 individuals from the federal state of Thuringia during the 2014/2015 season highlighted key factors influencing repeated vaccination. Higher vaccination rates were observed among nursing home residents, individuals with chronic conditions, and older adults, while women, those with lower care levels, and individuals with comorbidities were less frequently vaccinated. Additionally, participation in disease management programs was associated with increased vaccination frequency. These findings underscore the importance of targeted vaccination strategies, especially for vulnerable populations, to improve consistent uptake [37].

Comprehensive strategies to enhance influenza vaccination rates have demonstrated tangible outcomes. A study by Saß et al. (2008) showed that members of different social classes were reached equally [38]. The survey also indicated that the majority of respondents (86%) followed medical recommendations and were regularly reminded of their vaccination status by their treating physician (84%), which significantly positively influenced vaccination rates.

In conclusion, the significance of medical counselling and education is apparent, with healthcare provider recommendation identified as the principal factor for vaccine uptake [34, 39]. Nevertheless, our study found that the uptake of pneumococcal and herpes zoster vaccination remains low in comparison to the high levels of uptake observed for the influenza vaccination. It is also important to note that the influenza vaccination only provides full protection if it is taken regularly at the recommended interval of one year [12, 13]. Consequently, regular education is of the utmost importance, as health literacy plays a significant role in determining vaccination barriers [40]. Our resident survey revealed that the most frequently cited barriers were the perceived lack of benefits (one-third of respondents), absence of a sense of belonging to a risk group (one-quarter), and fear of side effects. These barriers were significantly associated with non-uptake of influenza, pneumococcal, and herpes zoster vaccination, aligning with barriers reported in other studies [34, 41].

These findings indicate a critical gap in health literacy, particularly regarding risk group awareness among the ageing population over 60. Despite STIKO recommendations for age-specific vaccinations in this group, given that influenza and pneumococcal infections substantially increase the risk of complications and mortality in individuals with multiple health conditions, many older adults do not perceive themselves as at-risk, representing a key barrier to vaccination uptake [42, 43].

Upon reflection on the factors influencing the utilisation of these services, the pivotal role of general practitioners in providing them becomes evident. This, in turn, necessitates a high level of interest and, above all, capacity within the practices. In view of the significant undersupply in the more rural federal states, such as ST, and the resulting limited medical infrastructure, it is currently not possible to provide general practitioners in the long term [44]. In conclusion, it is important to emphasise that there should be transparent communication of benefits and risks, regardless of educational status [45].

Several limitations should be considered. The claims data were restricted to 2020 and excluded privately insured individuals (about 5% of the total population), while post-pandemic vaccination patterns remain unexplored. The cross-sectional design precludes causal inferences. The resident survey exhibited selection bias toward health-conscious participants, likely contributing to overestimated self-reported vaccination rates compared to claims data. Social desirability and recall bias may have further influenced responses. Spatial clustering analyses were not conducted; future research could employ such methods to better understand geographic determinants of uptake especially below county level if these data is available (not at the Zi). Finally, findings may not generalize beyond the selected urban and rural municipalities in Saxony-Anhalt.

## Conclusion

Beyond study limitations and despite favorable vaccination attitudes in Saxony-Anhalt, we recommend targeted education as essential to improve pneumococcal and herpes zoster uptake. Physician recommendation emerged as a key facilitator [34, 39, 46]. Germany’s existing infrastructure—statutory health insurer reminder systems (§ 20d SGB V) and Federal Centre for Health Education campaigns—should be adapted to target these vaccines through age-tailored messaging combined with GP reminder systems and shared decision-making tools [47]. Combined GP and medical assistant training on communication and organizational processes warrants further evaluation [48]. In rural settings with limited provider density, standing order protocols, practice-level audit and feedback, and performance-based reimbursement could reduce GP workload while maintaining quality [49].

Future research starting from our explanatory study should prioritize: (1) standardized recall systems coordinated between statutory health insurers and primary care; (2) local public health office roles in supporting GP-based vaccination delivery in underserved areas; (3) registry-linkage studies addressing reporting biases; and (4) multicomponent interventions adapted to regional contexts. Health literacy interventions embedded within GP consultations and insurer communications could improve self-assessment accuracy and vaccination awareness, reducing the gap between actual and perceived coverage.

## Appendix

**Appendix A:** Sample characteristics of the Zi data from Saxony-Anhalt

	2011	2012	2013	2014	2015	2016	2017	2018	2019	2020
**Total (N) aged 60 and over**	722,618	728,112	734,558	738,086	741,557	744,753	749,213	752,523	756,159	760,868
**age**										
60–64 65–69 70–74 75–79 80–84 85–89 90–94 ≥ 95	143,236 128,666 174,765 122,678 84,635 46,909 17,472 4,257	151,487 121,422 170,232 129,156 84,774 47,638 19,610 3,793	160,073 115,629 164,099 137,188 83,475 49,855 20,791 3,448	163,103 114,718 153,792 143,293 86,604 51,443 21,259 3,874	163,250 124,948 135,029 148,590 90,345 53,142 21,449 4,804	162,800 136,146 117,980 151,713 95,370 53,709 21,585 5,450	161,041 144,306 111,860 148,382 101,307 54,223 22,142 5,952	159,477 152,141 106,237 143,435 108,179 53,667 23,173 6,214	159,640 155,132 105,763 134,843 113,948 56,326 24,092 6,415	160,022 155,639 115,558 118,712 118,837 60,009 25,255 6,836
**sex**										
men women	302,667 419,951	306,301 421,811	310,598 423,960	313,087 424,999	315,519 426,038	317,386 427,367	319,939 429,274	322,032 430,491	324,194 431,965	326,515 434,353
**region**										
Wanzleben-Börde Halle (Saale) Magdeburg Sangerhausen Germany	48,988 69,989 70,756 50,605 13,997,848	49,999 70,021 71,029 50,679 14,106,724	50,968 70,392 71,662 51,046 14,188,752	51,619 70,516 72,302 51,327 14,248,115	52,400 70,521 72,453 51,675 14,263,658	52,983 70,384 72,710 51,962 14,351,817	53,636 70,477 72,750 52,123 14,490,877	54,357 70,155 72,660 52,073 14,825,404	55,256 70,263 72,605 52,300 14,725,939	56,138 70,285 72,692 52,764 14,859,577

**Appendix B:** Sample characteristics of the resident survey

	Men		Women		Total	
	*n*	%	*n*	%	*n*	%
**Number (N)** of participants aged 60 and over	441		423		864	
**Age** (*n* = 864)						
60 to 64 years 65 to 69 years 70 to 74 years 75 to 79 years 80 to 84 years 85 to 89 years 90 to 94 years 95 years and over	46 54 58 71 59 100 27 26	10.4 12.2 13.2 16.1 13.4 22.7 6.1 5.9	55 65 68 59 61 63 17 35	13 15.4 16.1 13.9 14.4 14.9 4 8.3	101 119 126 130 120 163 44 61	11.7 13.8 14.6 15 13.9 18.9 5.1 7.1
**Region** (*n* = 778)						
Sangerhausen Halle (Saale) Wanzleben-Börde Magdeburg	77 95 102 115	19.8% 14.4% 26.2% 29.6%	83 81 94 131	21.3% 20.8% 24.2% 33.7%	160 176 196 246	20.6% 22.6% 25.2% 31.62%
**Educational Level** (*n* = 823)						
low medium high	47 200 174	11.2% 47.5% 41.3%	68 243 91	16.9% 60.4% 22.5%	115 443 265	14% 53.8% 32.2%
**Partnership Status** (*n* = 856)						
Yes	357	81.5%	243	58.1%	600	70.1%
No	81	18.5%	175	41.9%	256	29.9%
**Monthly Net Household Income** (*n* = 724)						
< 500€ 500€ to < 1000€ 1000€ to < 2000€ 2000€ to < 3000€ 3000€ to < 5000€ ≥ 5000€	1 16 120 138 89 6	0.3% 4.3% 32.4% 37.3% 24.1% 1.6%	1 30 148 127 44 4	0.3% 8.5% 0.4% 35.9% 12.4% 1.1%	2 46 268 265 133 10	0.3% 6.4% 37% 36.6% 18.4% 1.4%

Note. The remaining participants (under 60 years) make up the remainder of the original dataset (*N* = 954)

**Appendix C:** From Saxony-Anhalt

	2011	2012	2013	2014	2015	2016	2017	2018	2019	2020
**Total (N) aged 60 and over**	722,618	728,112	734,558	738,086	741,557	744,753	749,213	752,523	756,159	760,868
**a**										
	143,236 128,666 174,765 122,678 84,635 46,909 17,472 4,257	151,487 121,422 170,232 129,156 84,774 47,638 19,610 3,793	160,073 115,629 164,099 137,188 83,475 49,855 20,791 3,448	163,103 114,718 153,792 143,293 86,604 51,443 21,259 3,874	163,250 124,948 135,029 148,590 90,345 53,142 21,449 4,804	162,800 136,146 117,980 151,713 95,370 53,709 21,585 5,450	161,041 144,306 111,860 148,382 101,307 54,223 22,142 5,952	159,477 152,141 106,237 143,435 108,179 53,667 23,173 6,214	159,640 155,132 105,763 134,843 113,948 56,326 24,092 6,415	160,022 155,639 115,558 118,712 118,837 60,009 25,255 6,836
**sex**										
men women	302,667 419,951	306,301 421,811	310,598 423,960	313,087 424,999	315,519 426,038	317,386 427,367	319,939 429,274	322,032 430,491	324,194 431,965	326,515 434,353
**region**										
Wanzleben-Börde Halle (Saale) Magdeburg Sangerhausen Germany	48,988 69,989 70,756 50,605 13,997,848	49,999 70,021 71,029 50,679 14,106,724	50,968 70,392 71,662 51,046 14,188,752	51,619 70,516 72,302 51,327 14,248,115	52,400 70,521 72,453 51,675 14,263,658	52,983 70,384 72,710 51,962 14,351,817	53,636 70,477 72,750 52,123 14,490,877	54,357 70,155 72,660 52,073 14,825,404	55,256 70,263 72,605 52,300 14,725,939	56,138 70,285 72,692 52,764 14,859,577
